# Assessment of EuroSCORE II and STS Score Performance and the Impact of Surgical Urgency in Isolated Coronary Artery Bypass Graft Surgery at a Referral Center in São Paulo, Brazil

**DOI:** 10.21470/1678-9741-2023-0282

**Published:** 2024-09-03

**Authors:** Plínio José Whitaker Wolf, Vivian Lerner Amato

**Affiliations:** 1 Department of Coronary Artery Disease, Instituto Dante Pazzanese de Cardiologia, São Paulo, São Paulo, Brazil

**Keywords:** Heart Disease Risk Factors, Risk Assessment, Myocardia Revascularization, Coronary Disease

## Abstract

**Introduction:**

Risk prediction models, such as The Society of Thoracic Surgeons (STS) risk score and the European System for Cardiac Operative Risk Evaluation II (EuroSCORE II), are recommended for assessing operative mortality in coronary artery bypass grafting (CABG). However, their performance is questionable in Brazil.

**Objective:**

To assess the performance of the STS score and EuroSCORE II in isolated CABG at a Brazilian reference center.

**Methods:**

Observationaland prospective study including 438 patients undergoing isolated CABG from May 2022-May 2023 at the Instituto Dante Pazzanese de Cardiologia. Observed mortality was compared with predicted mortality (STS score and EuroSCORE II) by discrimination (area under the curve [AUC]) and calibration (observed/expected ratio [O/E]) in the total sample and subgroups of stable coronary artery disease (CAD) and acute coronary syndrome (ACS).

**Results:**

Observed mortality was 4.3% (n=19) and estimated at 1.21% and 2.74% by STS and EuroSCORE II, respectively. STS (AUC=0.646; 95% confidence interva [CI] 0.760-0.532) and EuroSCORE II (AUC=0.697; 95% CI 0.802-0.593) presented poor discrimination. Calibration was absent for the North American mode (P<0.05) and reasonable for the European model (O/E=1.59, P=0.056). In the subgroups, EuroSCORE II had AUC of 0.616 (95% CI 0.752-0.480) and 0.826 (95% CI 0.991-0.661), while STS had AUC of 0.467 (95% CI 0.622-0.312) and 0.855 (95% CI 1.0-0.706) in ACS and CAD patients, respectively, demonstrating good score performance in stable patients.

**Conclusion:**

The predictive models did not perform optimally in the total sample, but the EuroSCORE was superior, especially in elective stable patients, where accuracy was satisfactory.

## INTRODUCTION

Coronary artery disease (CAD) is the leading cause of death in Brazil^[1]^, and coronary artery bypass grafting (CABG) is the treatment of choice for many patients with severe CAD, being the most frequent cardiac surgery in this country^[2]^ and worldwide (55% of cardiac surgeries according to data from large centers)^[3]^. Due to its high prevalence and inherent risks, operative risk assessment is essential.

Risk prediction models, developed to estimate morbidity and mortality outcomes in cardiovascular surgeries, are widely used tools that offer significant assistance to health services, public policies, and medical management. In this context, we highlight two risk prediction models with recognized accuracy that are recommended by current international guidelines^[4,5]^ and are intensively used in the medical community: The Society of Thoracic Surgeons (STS) risk score and the European System for Cardiac Operative Risk Evaluation II (EuroSCORE II).

The North American STS score model, created in the 1990s and last updated (2018) from a sample of 439,092 surgeries^[6]^, takes into account 65 complex variables, assessing operative mortality, as well as eight morbidity-related outcomes^[7]^. Easier to manage (composed of 18 risk factors), the EuroSCORE II, developed in 2011 from 22,381 patients, assesses only operative mortality^[8]^. Both models have satisfactory performance in predicting mortality in the populations in which they were developed^[6,7,8,9]^, but studies suggest superiority of the North American model^[10,11]^.

However, they were developed and validated predominantly in a population with different characteristics from the Brazilian reality and other countries. In regard to isolated CABG, the mortality observed by the STS registries in 2019 was 2.2%^[12]^, while in Brazil, between 2005 and 2007, it was 6.22%^[2]^, confirming these inequalities.

In accordance with this rationale, a study conducted in Turkey in 2013 compared EuroSCORE II, EuroSCORE, and STS in isolated CABG, showing that the first score underestimated mortality, estimated at 1.7% and observed at 7.9%^[13]^. Also, in a retrospective Brazilian study published in 2020 evaluating risk scores within a sample of 5,222 cardiac surgeries, Mejía OAV et al.^[14]^ observed a mortality (7.6%) much higher than the one estimated by the European (3.1%) and North American (1.0%) models.

It is evident, therefore, that the performance of a predictive model differs according to the population groups in which it is applied, making it necessary to assess its accuracy in the Brazilian population, characterized by specific clinical presentations, given the deep socioeconomic and cultural differences, as well as in the distribution and access to health services^[15,16]^.

Thus, this study aims to assess the performance in predicting operative mortality of the two main risk models currently used and recommended, STS score and EuroSCORE II, regarding isolated myocardial revascularization surgeries, in a reference center in Brazil, the Instituto Dante Pazzanese de Cardiologia (IDPC).

## METHODS

This is an observational, prospective, single-center study conducted by collecting data from patients undergoing isolated CABG at IDPC.

### Data Selection

All of the variables used to calculate the risk of mortality by the risk models analyzed in this study, the STS score (65 variables) and EuroSCORE II (18 variables), were obtained prior to surgery, in order to subsequently estimate the operative risk of patients using the two models above mentioned^17,19^.

The outcome assessed was operative mortality, defined as death occurring during the surgical hospitalization (regardless of the length of hospital stay) or occurring within 30 days after the surgical procedure, if discharged from hospital before this period.

In addition to these data, educational level and glycated hemoglobin (variables not present in the scores) were collected, for further characterization of the study population.

### Variable Definition

The definition of each variable selected and collected is aligned exactly with the recommendations of the EuroSCORE II^[8]^ and STS score^[19]^ predictor models.

Creatinine clearance was calculated using the Cockcroft-Gault formula (the same as in EuroSCORE II).

Among the data collected, the variables “Canadian Cardiovascular Society IV (CCS IV) angina”, “extracardiac arteriopathy“, “poor mobility”, “recent infarction”, and “critical preoperative state” are exclusive to the EuroSCORE II model, while “cerebrovascular disease”, “heart failure (HF)” and “immunosuppression” are specific to the STS score. The definition and classification of surgical urgency are similar in both scores and were adopted in this study (elective: performed in routine admissions and can be delayed without causing additional cardiac risk; urgency: patient was not electively admitted for the procedure, which must be performed in the same hospitalization; emergency: must occur before the start of the next working day; salvage: under cardiopulmonary resuscitation). However, “chronic lung disease” and “previous cardiac surgery” are variables present in both scores, but with different definitions. In this study, the specific definition of each variable was adopted for the calculation of the respective score, but for the description of the characteristics of the overall sample, “chronic lung disease” was defined according to EuroSCORE II (necessity of bronchodilator use) and “previous cardiac surgery” according to STS (any cardiac procedure, including percutaneous coronary intervention [PCI]).

### Sample Selection/Casuistry

Patients selected for the sample and data collection were all those admitted to the IDPC with scheduled isolated CABG in the same hospitalization, regardless of clinical status, degree of urgency, or surgical indication. Patients undergoing isolated CABG from May 2022 to May 2023 were prospectively included. If there was another associated surgical procedure during the operation, the patient was excluded.

### Statistical Analysis

Continuous variables were described by their means and standard deviations. Categorical variables were described using absolute and relative frequencies.

Initial comparisons of baseline characteristics were performed by Fisher’s exact tests (categorical variables) or Student’s *t*-tests (continuous variables).

The results concerning observed and predicted mortality by the risk models were analyzed to determine the performance of EuroSCORE II and STS score (predictive validation of the models), by calibration (assessed by the observed to predicted mortality ratio, with satisfactory calibration when *P*>0.05) and discrimination (assessed by the area under the curve [AUC] of the receiver operating characteristic [ROC] curve, being adequate when closer to 1.0 and absent if < 0.5) tests.

Calibration of models was also assessed within established risk ranges of predicted mortality (≤ 3%: low risk; from 3 to 6%: moderate risk; ≥ 6%: high risk) and in specific subgroups of stable CAD and acute coronary syndrome (ACS), which included unstable angina and acute myocardial infarction.

Results were expressed with 95% confidence intervals (CI).

Analyses were conducted using R software, version 4.2.1.

### Ethical Considerations

The study complied with all required ethical principles and was approved by the IDPC Research Ethics Committee (Research Ethics Committee number: 5244; report number: 5.383.227; Certificate of Submission for Ethical Assessment: 57362122.7.0000.5462). Patients had access to a Free and Informed Consent Form, in accordance with the general data protection law and resolution 466/2012.

## RESULTS

### Baseline Characteristics of the Sample and Subgroups

The study included 438 patients who underwent isolated CABG in the one-year period evaluated. The mean age was 62 ± 8.2 years (17.6% were older than or aged 70 years), 26.5% were women, and 76.7% were Caucasian. Table 1 shows the baseline characteristics regarding the clinical background of the sample.

**Table 1:** Baseline clinical characteristics of the sample (n=438).

Variable	Total (n=438)
Age, mean ± SD	62.0 ± 8.2
Age ≥ 70 years	77/438 (17.6%)
Sex, male	322/438 (73.5%)
Ethnicity	
Caucasian	336/438 (76.7%)
Brown	74/438 (16.9%)
Black	28/438 (6.4%)
Educational level	
Illiterate	13/265 (4.9%)
Elementary school	83/265 (31.3%)
Middle school	51/265 (19.2%)
High school	88/265 (33.2%)
University education	30/265 (11.3%)
BMI (kg/m^2^)	27.9 ± 4.7 (n = 438)
BSA (m^2^)	1.91 ± 1.15 (n = 434)
SAH	395/438 (90.2%)
DM	236/438 (53.9%)
IDDM	138/438 (31.5%)
Illicit drugs	6/438 (1.4%)
Alcoholism	
No	412/438 (94.1%)
Former alcoholic	15/438 (3.4%)
Alcoholic	11/438 (2.5%)
Smoking	
No	187/438 (42.6%)
Yes	251/438 (57.3%)
Former smoker	169/438 (38.5%)
Current smoker	82/438 (18.7%)
Extracardiac arteriopathy*	106/438 (24.2%)
PAD	64/438 (14.6%)
Cerebrovascular disease^†^	81/438 (18.5%)
Previous stroke	24/430 (5.6%)
Carotid disease (> 50%)	
Present	56/438 (12.7%)
Unilateral	36/438 (8.2%)
Bilateral	20/438 (4.6%)
Poor mobility	18/438 (4.1%)
Chronic lung disease	27/438 (6.2%)
Immunocompromised	7/438 (1.6%)
Family history of CAD	48/438 (11.0%)
Prior PCI	55/438 (12.5%)

BMI=body mass index; BSA=body surface area; CAD=coronary artery disease; DM=diabetes mellitus; IDDM=insulin-dependent diabetes mellitus; PAD=peripheral artery disease; PCI=percutaneous coronary intervention; SAH=systemic arterial hypertension; SD=standard deviation

* carotid stenosis, amputation, approach to aneurysm of the abdominal aorta or other arteries

† stroke, transient ischaemic attack, carotid stenosis, previous approach carotid stenosis

Table 2 correlates the baseline characteristics of hospitalization with the indication for CABG (stable CAD or ACS subgroups), showing a higher prevalence of surgical indication for patients with ACS compared with stable CAD (64.2% and 35.8%, respectively) and, therefore, urgent and emergency surgeries were the most frequent surgical statuses.

**Table 2:** Comparison of baseline characteristics (n=438) at admission (clinical, laboratory, echocardiographic, and anatomical) according to indication for coronary artery bypass grafting (stable coronary artery disease and acute coronary syndrome subgroups).

Variable	Stable CAD (n = 157)	ACS (n = 281)	Total	*P*-value*
AMI < 90 days	0/157 (0.0%)	201/281 (71.5%)	201/438 (45.9%)	< 0.001
Time since AMI (days)	146.5 ± 48.2 (n = 20)	30.0 ± 35.0 (n = 213)	40.0 ± 48.8 (n = 233)	< 0.001
CCS IV angina	0/157 (0.0%)	49/281 (17.4%)	49/438 (11.2%)	< 0.001
Coronary anatomy				0.861
One-vessel disease	3/157 (1.9%)	3/281 (1.0%)	6/438 (1.3%)	
Two-vessel disease	15/157 (9.5%)	30/281 (10.6%)	45/438 (10.2%)	
Three-vessel disease	94/157 (59.8%)	157/281 (55.8%)	251/438 (57.3%)	
LMD > 50%	45/157 (28.7%)	91/281 (32.4%)	136/438 (31.1%)	0.452
Heart failure	41/157 (26.1%)	113/281 (40.2%)	154/438 (35.1%)	0.006
Ejection fraction (%)	54.3 ± 10.3 (n = 157)	49.8 ± 12.0 (n = 281)	51.4 ± 11.7 (n = 438)	< 0.001
Ejection fraction				
< 30%	5/157 (3.2%)	14/281 (5.0%)	19/438 (4.3%)	0.003
30 - 50%	37/157 (23.6%)	107/281 (38.1%)	144/438 (32.9%)	
≥ 50%	115/157 (73.2%)	160/281 (56.9%)	275/438 (62.8%)	
NYHA functional class				
1	23/142 (16.2%)	41/219 (18.7%)	64/361 (17.7%)	< 0.001
2	86/142 (60.6%)	71/219 (32.4%)	157/361 (43.5%)	
3	32/142 (22.5%)	89/219 (40.6%)	121/361 (33.5%)	
4	1/142 (0.7%)	18/219 (8.2%)	19/361 (5.3%)	
Pulmonary hypertension^†^	44/157 (28.0%)	96/281 (34.2%)	140/438 (32.0%)	0.201
PASP > 55 mmHg	3/28 (10.7%)	4/70 (5.7%)	7/98 (7.1%)	0.404
Critical preoperative state^‡^	1/157 (0.6%)	9/281 (3.2%)	10/438 (2.3%)	0.104
Urgency of operation				
Elective	145/157 (92.4%)	2/281 (0.7%)	147/438 (33.6%)	< 0.001
Emergency	0/157 (0.0%)	8/281 (2.8%)	8/438 (1.8%)	
Urgent	12/157 (7.6%)	271/281 (96.4%)	283/438 (64.6%)	
Complementary exams				
Haematocrit (%)	41.4 ± 5.0	39.3 ± 5.6	40.0 ± 5.4	< 0.001
Leukocytes (1000/mm^3^)	7605.2 ± 2087.5	7952.7 ± 2183.9	7828.2 ± 2153.9	0.101
Platelet count (1000/mm^3^)	196461.5 ± 50302.4	217622.8 ± 67127.9	210068.6 ± 62421.4	< 0.001
Glycated haemoglobin (%)	6.82 ± 1.87	6.83 ± 1.74	6.83 ± 1.79	0.975
Serum creatinine (mg/dL)	1.04 ± 0.38	1.03 ± 0.45	1.04 ± 0.42	0.908
Clearance (mlVm¡n)^§^	84.7 ± 29.6	84.9 ± 29.8	84.8 ± 29.7	0.923
Haemodialysis	1/157 (0.6%)	4/281 (1.4%)	5/438 (1.1%)	0.659

ACS=acute coronary syndrome; AMI=acute myocardial infarction; CAD=coronary artery disease; CCS=Canadian Cardiovascular Society; LMD=left main disease; NYHA=New York Heart Association; PASP=pulmonary artery systolic pressure

* Fisher’s exact test for categorical variables, presented as n/N (%), and Student’s *t*-tests for continuous variables, represented as mean and standard deviation

† Pulmonary hypertension defined by PASP > 30 mmHg

‡ Preoperative critical state: intra-aortic balloon, anuria/oliguria, inotropic, cardiopulmonary arrest, and preoperative invasive ventilation

§ Calculated by the Cockcroft-Gault formula

### Outcome and Performance Assessment of Predictor Models

The observed operative mortality was 4.3% (19 patients). All deaths occurred during hospitalization (no outcome occurred after discharge, within 30 days). Estimated operative mortality was 2.74% and 1.21% according to the EuroSCORE II and STS score models, respectively.

Tables 3 4 demonstrate the calibration of EuroSCORE II and STS, respectively, by analyzing the observed/expected ratio (O/E), which is optimal when closer to 1.0 and positive when the *P*-value is > 0.05.

**Table 3:** EuroSCORE II calibration in the total sample, in the subgroups according to the indication for myocardial revascularization (stable coronary artery disease and acute coronary syndrome) and according to the risk ranges.

Group	Observed mortality	Predicted mortality (%)	Observed/expected ratio	*P*-value
Total (n=438)	19/438 (4.3%)	2.74	1.59	0.056
Diagnosis				
Stable CAD (n=157)	5/157 (3.2%)	1.83	1.74	0.140
ACS (n=281)	14/281 (5.0%)	3.24	1.54	0.331
Risk ranges				
< 3.00%	10/333 (3.0%)	1.51	1.99	
3.00 – 6.00%	7/71 (9.9%)	4.1	2.41	
≥ 6.00%	2/34 (5.9%)	11.9	0.49	

ACS=acute coronary syndrome; CAD=coronary artery disease; EuroSCORE II = European System for Cardiac Operative Risk Evaluation II *P*-value for test of adherence of observed mortality to predicted mortality

*P*-value for test of adherence of observed mortality to predicted mortality

**Table 4:** Calibration of the Society of Thoracic Surgeons score in the total sample, in subgroups according to the indication for coronary artery bypass grafting (stable coronary artery disease and acute coronary syndrome) and according to risk ranges.

Group	Observed mortality	Predicted mortality (%)	Observed/expected ratio	*P*-value
Total (n=438)	19/438 (4.3%)	1.21	3.60	< 0.001
Diagnosis				
Stable CAD (n=157)	5/157 (3.2%)	0.871	3.66	< 0.001
ACS (n=281)	14/281 (5.0%)	1.39	3.58	0.007
Risk ranges				
< 3.00%	19/416 (4.6%)	1.03	4.45	
3.00 – 6.00%	0/18 (0%)	3.95	-	
≥ 6.00%	0/4 (0%)	7.5	-	

ACS=acute coronary syndrome; CAD=coronary artery disease

*P*-value for test of adherence of observed mortality to predicted mortality

Calibration was assessed in the specific subgroups of stable CAD and ACS, whose observed mortalities in the study population were 3.2% and 5.0%, respectively.

Table 3 reveals that, despite not being ideal (O/E=1.59), the calibration of the European score was positive in the analyzed sample (*P*>0.05). When assessing the calibration in the subgroups of CAD and ACS patients, it remained positive (with *P*-values higher than the calibration of the total sample), however, when assessing its performance according to risk ranges, there is a loss of calibration at high risks (> 6%), with a predicted mortality of 11.9% and an observed death of 5.9%.

Table 4, in turn, elucidates an absent calibration of the STS score in this sample, with a *P*-value < 0.001 and an O/E > 3.5 in all subgroups evaluated, including stable CAD and ACS and all established risk ranges, which makes its European competitor superior in this regard.

**Fig. 1:**
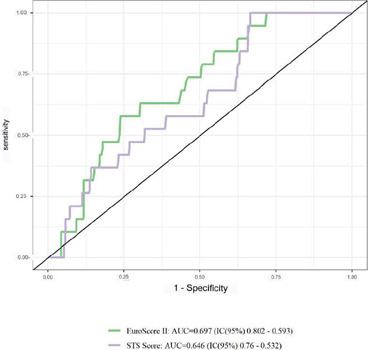
Receiver operating characteristic curve for mortality according to the European System for Cardiac Operative Risk Evaluation II (EuroSCORE II) and the Society of Thoracic Surgeons (STS) score for assessment of discrimination capacity in the total sample (n=438). AUC=area under the curve; CI=contidence interval.

**Fig. 2:**
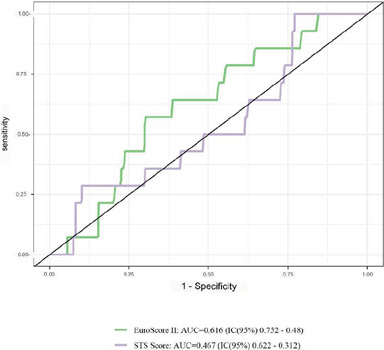
Receiver operating characteristic curve for mortality according to the European System for Cardiac Operative Risk Evaluation II (EuroSCORE II) and the Society of Thoracic Surgeons (STS) score for assessment of discrimination capacity in the subgroup of patients admitted with acute coronary syndrome (n=281). AUC=area under the curve; CI=confidence interval.

**Fig. 3:**
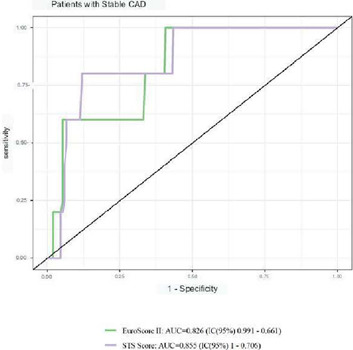
Receiver operating characteristic curve for mortality according to the European System for Cardiac Operative Risk Evaluation II (EuroSCORE II) and the Society of Thoracic Surgeons (STS) score for assessment of discrimination capacity in the subgroup of patients admitted with stable coronary artery disease (n=157). AUC=area under the curve; CI=confidence interval.

Discrimination was assessed both in the total sample and in the stable CAD and ACS subgroups using the ROC curve, as illustrated in (Figures 1, 2, 3).

Figure 1 demonstrates the area under the ROC curve in the total sample and reveals positive discrimination (AUC > 0.5) for the STS score (AUC=0.646; 95% CI 0.760-0.532) and EuroSCORE II (AUC=0.697; 95% CI 0.802-0.593); however, due to AUC values < 0.75, poor discrimination is observed.

Nevertheless, while discrimination of the models was very limited in the subgroup of patients admitted due to ACS, as shown in Figure 2 (AUC=0.616 and AUC=0.467 for EuroSCORE II and STS score, respectively), discrimination in patients with stable CAD was highly positive. In this subgroup of individuals with stable CAD, the AUC was > 0.8 for the European model (AUC=0.826; 95% CI 0.991-0.661) and for the North American model (AUC=0.855; 95% CI 1.0-0.706), as illustrated in Figure 3.

## DISCUSSION

### Analysis of the Observed Operative Mortality Outcome

This is an important prospective study to assess the performance of risk prediction models that are most recommended by current guidelines in a large cardiac surgery referral center in Brazil.

The operative mortality in the analyzed center (4.3%), which is part of the Brazilian public health system (Sistema Único de Saúde [SUS]), proved to be lower than that reported by other previous studies in Brazil, whose mortality for CABG is around 6.22%^[2]^, ranging from 5% to 9.4% according to the center evaluated^[20^, ^21]^. In fact, there was evidence of a reduction in operative mortality in CABG in the service evaluated, since a previous retrospective analysis (1999-2017) showed a rate of 5%^[22]^. Even so, the mortality rate is still higher than the reported rates in developed countries, which are around 1.8 to 2.7%^[11,23]^.

As the predictor models, EuroSCORE II and STS, were developed in databases of populations whose mortality is much lower than those of underdeveloped countries, there is a loss of accuracy of the scores when applied to other samples, as shown by previous studies^[13]^, including in Brazilian centers^[14,15,16]^. However, in Brazil, the healthcare system is marked by inequality and heterogeneity and, hence, the performance of the two main predictor models at the IDPC was not known. This study, therefore, demonstrated that accuracy was poor for both predictor models (especially for the North American model) in this population as a whole.

### Analysis of the Performance of Predictor Models (EuroSCORE II and STS Score)

The EuroSCORE II was found to be superior to the STS score, which is the opposite of what large studies describe^[11]^ and what guidelines recommend^[4,5]^. While the European model has a positive calibration (O/E=1.59, with *P*>0.056), the North American score was uncalibrated (O/E=3.6, with *P*<0.001). Furthermore, the discrimination of the EuroSCORE II (AUC=0.697), although not ideal, is still better than of the STS score (AUC=646). This is probably due to the assessed tendency of the STS score to underestimate operative risk and, in a sample where mortality appears to be slightly higher, estimating a lower risk decreases its accuracy. Other studies have also shown that this model generally provides a lower estimated risk than the European concurrent^[10,11,14]^.

There are numerous factors that may be related to the increase in operative mortality in the analyzed population with consequent loss of performance of the predictive models. Socioeconomic, cultural, and geographic factors may be associated, but it is not known objectively how these issues have led to increased mortality in this and other studies.

It is noted that most of the baseline characteristics of the patients in this study are similar to the variables of other populations found in developed countries, as shown in Table 5, composed by this study population (n=438), EuroSCORE II sample (n=22,381, recruited in 2010), a relevant study conducted in Italy by Paparella D et al.^[24]^ (n=6,293, analyzed with data from the Puglia Adult Cardiac Surgery Registry from 2011 to 2012, which assessed the accuracy of EuroSCORE II in operative mortality), and STS analysis published in 2009 (n=774,881, evaluated from 2002 to 2006, in order to update and validate the STS score in that year)^[8^, ^9]^. Variables related to age, gender, renal function, peripheral arterial disease, and functional class were quite similar. Interestingly, however, the samples differed greatly in terms of surgical urgency. While in the EuroSCORE II, STS score, and Paparella D et al.^[24]^ survey populations urgent/emergency surgery was indicated in 22.8%, 19.1%, and 50.3% respectively, our center included 66.4% of patients with urgent surgery. Also, the prevalence of recent infarction was more frequent in our sample, corresponding to 45.9% compared to 16.8% in the study conducted in Italy. In the three foreign analyses (EuroSCORE II, Paparella D et al.^[24]^, and STS score 2009), the predictive models performed well (discrimination represented by AUC > 0.8), which was not the case in our study.

**Table 5:** Comparison of baseline characteristics of this study (n=438) with the sample of EuroSCORE II (n=22,381), Paparella et al. (n=6,293), and the STS published in 2009 (n=774,881).

Variable	Frequency or mean in IDPC sample (n=438)	Frequency or mean in the EuroSCORE II sample (n=22,381)*	Frequency or mean in the STS 2009 sample (n=774,881)^†^	Frequency or mean in Paparella et al. sample (n=6293)^‡^
Age, mean ± SD (years)	62.0 ± 8.2	64.6 ± 12.5	NR	67.4 ± 11.2
Sex, female	26.4%	30.9%	27.7%	34.1%
Serum creatinine (mg/dL)	1.04 ± 0.42	1.04 ± 0.42	NR	NR
Clearance (mL/min)	84.8 ± 29.7	83.6 ± 50.9	NR	76.3 ± 31.5
Haemodialysis	1.1%	1.1%	1.6%	1.4%
BMI (kg/m^2^)	27.9 ± 4.7	27.4 ± 4.8	NR	27.5 ± 4.4
PAD	14.6%	NR	15.5%	NR
Chronic lung disease	6.2%	10.7%	21%	9.2%
**IDDM**	**31.5%**	**7.6%**	**10.89%**	**10%**
NYHA functional class				
I	17.7%	NR	12.6%	25.2%
II	43.5%	NR	24.2%	37.1%
III	33.5%	NR	37.1%	34.8%
IV	5.3%	NR	21.3%	5.6%
CCS IV angina	11.2%	NR	NR	5.6%
**AMI < 90 days**	**45.9%**	**NR**	**NR**	**16.8%**
Ejection fraction (%)	51.4 ± 11.7	NR	NR	NR
< 30%	4.3%	NR	NR	5.4%
30-50%	32.9%	NR	NR	42.9%
> 50%	62.8%	NR	NR	56.4%
Pulmonary hypertension	32%	NR	NR	18.2%
PASP > 55 mmHg	7.1%	NR	NR	4.7%
Urgency of operation				
Elective	33.6%	76.7%	49.1%	80.6%
**Urgent/emergency**	**66.4%**	**22.8%**	**50.3%**	**19.1%**
Isolated CABG	100%	46.7%	100%	42%
Observed mortality rate	4.3%	4.15%	2.3%	4.85%
EuroSCORE II (AUC)^§^	2.74% (0.697)	3.9% (0.809)	NR	4.4% (0.830)
STS score (AUC)^§^	1.21% (0.646)	NR	NR (0.812)	NR

AMI=acute myocardial infarction; AUC=area under the curve; BMI=body mass index; CABG=coronary artery bypass grafting; CCS=Canadian Cardiovascular Society; EuroSCORE II=European System for Cardiac Operative Risk Evaluation II; IDDM=insulin-dependent diabetes mellitus; IDPC=Instituto Dante Pazzanese de Cardiologia; NYHA=New York Heart Association; PAD=peripheral artery disease; PASP=pulmonary artery systolic pressure; NR=not reported; SD=standard deviation; STS=Society of Thoracic Surgeons

Pulmonary hypertension defined when PASP > 30 mmHg

Creatinine clearance calculated according to the Cockroft-Gault formula

* Sample from the study that developed and validated EuroSCORE II

† STS sample published in 2009 to update and validate the STS score that year

‡ Paparella et al. sample with data collected from the Puglia Adult Cardiac Surgery Registry

§ Discrimination of the predictor model by AUC

In this sense, it is realized that the fact that our service performs a large part of urgent/emergency surgeries may be associated with an increment in mortality and discrepancy in the prediction of risk models. This reflects Brazilian socioeconomic inequalities, which do not provide comprehensive primary care, leading to an increase in the prevalence of underdiagnosed and untreated comorbidities, prompting patients to request health services at an advanced stage of the disease, such as urgent cases of ACS.

Thus, it is evident that patients arriving with an indication for urgent surgery for an ACS are at higher risk. In the study, 73% of deaths occurred in patients admitted for ACS (an indication for urgent surgery), representing a mortality rate of 5% in this subgroup, in contrast to a mortality rate of 3.2% in patients with stable CAD. In addition to the pathophysiological mechanisms related to ACS (prothrombotic, inflammatory state, edema, and myocardial stunning), previous decompensated comorbidities diagnosed in an unplanned hospitalization contribute to a worse outcome. This is reflected in a high admission rate of diabetic patients (54% *vs.* 25% in EuroSCORE II), insulin-dependent diabetics (31.5% *vs.* 7.6% in EuroSCORE II)^[8]^, with pulmonary hypertension (32% *vs.* 18% in a study by Paparella D et al.^[24]^), showing a deficient primary care. Also, it can be observed that patients admitted in ACS have a higher prevalence of clinical criteria of severity, conferring higher operative mortality, as identified in Table 2. It is statistically significant (*P*<0.05) that ACS patients, compared with the chronic stable disease subgroup, are more often in CCS IV angina (17.4% *vs.* 0%), with worse New York Heart Association functional class (48.8% in class III/IV *vs.* 23.3%), lower ejection fraction (49.8% *vs.* 54.3%), and with HF (40.2% *vs.* 26.1%). In other words, in addition to conferring greater risk on its own, ACS is associated with other poor prognostic factors.

Because our service sample differs greatly from the populations that developed the predictor models regarding the prevalence of urgency and ACS, the performance of EuroSCORE II and STS score was evaluated in the subgroup of patients admitted only for stable CAD. In this case, a very good discrimination was observed for both models, reaching AUC > 0.8 (Figure 3), very similar to the values found in the studies in which they were validated™ The calibration of EuroSCORE II also proved to be adequate in this subgroup, with *P*=0.14.

On the other hand, when discrimination was assessed only in ACS patients (Figure 2), both scores were inadequate (AUC < 0.5). Thus, the high prevalence of ACS and the consequent surgical urgency contributed to increased mortality and inadequate scores performance in our service.

Therefore, it is feasible to state that, in view of the need to perform surgical risk in the center evaluated, EuroSCORE II is the most indicated score in our institution, since it has better calibration and discrimination. However, as previously stated, its performance is much better in patients with stable CAD (excellent discrimination and satisfactory calibration) and, in this population, the use of the European model seems to be reliable.

### Analysis of Other Sample Characteristics

The complexity of the coronary disease in the sample is particularly noteworthy, as 88.4% of patients undergoing CABG had three-vessel or severe left main lesions. This profile is similar to other databases, as shown by Shahian et al.^[9]^, in STS data published in 2009, where there were 75.7% and 28% of patients in the sample undergoing CABG with triple vessel disease and severe left main lesion, respectively. In our sample, few patients had one-vessel disease (1.3%), as well as in the mentioned STS analysis (4.17%), suggesting the likely greater indication for clinical treatment or PCI in this group of patients, leaving mainly those with more severe and complex disease for surgical revascularization.

Another important aspect demonstrated in this study is the reflection of the profile of patients using the public health system, since the center studied is an integral part of the SUS. It is observed that patients have a low educational level, with only 44.5% of individuals having completed high school, in addition to an illiteracy rate of 5%, in one of the most developed regions in Brazil. In parallel to educational access, the significant prevalence of comorbidities also reflects, as previously mentioned, socioeconomic difficulties in this population. Therefore, the research revealed a prominent prevalence of systemic arterial hypertension (90.2%), diabetes mellitus (53.9%), and an elevated body mass index (close to 28), comorbidities that are closely associated with a sedentary lifestyle, inadequate diet, and poor access to primary care, among other factors. For comparison, Shahian et al.^[9]^, in their data, showed rates of 78.3% and 36.4% for hypertension and diabetes, respectively, that is, diseases that are slightly less frequent in a sample with better socioeconomic indices.

### Limitations

The research had some limitations, particularly with regard to the small sample size, which may have an unfavorable impact on statistical power.

Finally, the evidence from this study that there is a notable prevalence of coronary urgent admissions suggests that it is only one but important factor contributing to the higher mortality and loss of performance of risk prediction models in the Brazilian population. Further studies are needed to confirm this hypothesis and also to search for other factors in our population and health services that may objectively justify the higher operative risk. After all, attributing, in a widespread and exclusive way, the socioeconomic aspect as the only etiology for a worse surgical outcome is comfortable and does not foster solutions for improvements in this context.

## CONCLUSION

The study showed that the main recommended risk prediction models (EuroSCORE II and STS score) did not perform optimally in the assessment of operative mortality of isolated CABG in the population of the IDPC, due to the tendency to underestimate the operative risk in the total sample evaluated. However, EuroSCORE II proved to be superior to the STS score, especially in patients admitted electively with stable CAD, where the model proved to be accurate.

## Abbreviations, Acronyms & Symbols

ACS: Acute coronary syndrome
AMI: Acute myocardial infarction
AUC: Area under the curve
BMI: Body mass index
BSA: Body surface area
CABG: Coronary artery bypass grafting
CAD: Coronary artery disease
CCS: Canadian Cardiovascular Society
CI: Confidence interval
DM: Diabetes mellitus
EuroSCORE II: European System for Cardiac Operative Risk Evaluation II
HF: Heart failure
IDDM: Insulin-dependent diabetes mellitus
IDPC: Instituto Dante Pazzanese de Cardiologia
LMD: Left main disease
NR: Not reported
NYHA: New York Heart Association
O/E: Observed/expected ratio
PAD: Peripheral artery disease
PASP: Pulmonary artery systolic pressure
PCI: Percutaneous coronary intervention
ROC: Receiver operating characteristic
SAH: Systemic arterial hypertension
SD: Standard deviation
STS: Society of Thoracic Surgeons
SUS: Sistema Único de Saúde
